# Characteristics of Type 2 Diabetes with Ketosis in Baoshan, Yunnan of China

**DOI:** 10.1155/2016/7854294

**Published:** 2016-01-10

**Authors:** Shichun Du, Xia Yang, Degang Shi, Qing Su

**Affiliations:** ^1^Department of Endocrinology, Xinhua Hospital Affiliated to Shanghai Jiaotong University School of Medicine, Shanghai 200092, China; ^2^Department of Endocrinology, Baoshan People's Hospital, Yunnan 678000, China

## Abstract

*Objectives*. The study provided data to demonstrate the characteristics of type 2 diabetes (T2D) with ketosis in rural parts of south-west border of China in order to help health professionals with optimizing diabetic care.* Methods*. All hospitalized adult diabetic patients consecutively between January 2011 and July 2015 in Baoshan People's Hospital, Yunnan province of China, were evaluated. T2D with ketosis, ordinary T2D (without ketosis), and type 1 diabetes (T1D) patients were analyzed according to the clinical and biochemical parameters and chronic complications in these subjects.* Results*. The prevalence of T2D with ketosis was 12% in the whole study subjects. Overweight and obese patients were predominant (49.1%) in T2D patients with ketosis. The mean HbA1c (13.3 ± 3.1%, *P* = 0.01), fasting plasma glucose (16.9 ± 6 mmol/L, *P* < 0.0001), and plasma triglyceride (4.0 ± 4.0 mmol/L, *P* < 0.0001) in T2D patients with ketosis were significantly higher than ordinary T2D patients without ketosis. Infections were the most common inducements in T2D patients with ketosis. Chronic complications including peripheral neuropathy (34.9%), retinopathy (12.7%), diabetic foot (18.1%), and persistent microalbuminuria (11.7%) were common in T2D patients with ketosis.* Conclusions*. This study indicated the poor glycemic control in diabetic patients in rural areas of south-west part of China. More efforts were urgently required to popularize public health education and improve medical quality in diabetic treatment in these regions.

## 1. Introduction

The outbreak of type 2 diabetes (T2D) is one of the largest public health problems around China [1–3]. Up till now, the prevalence of diabetes in adults of China has been 11.6% [2], while the current situations of diabetic control in rural areas are not clear. It is commonly acknowledged that diabetes is developing faster in urban areas than rural ones [2, 3]. However, the rural areas are becoming the disaster zone of diabetes due to the rapid changes in lifestyle with the development of economy and lack of adequate health education [4]. Many T2D patients are diagnosed with extreme hyperglycemia combined with ketosis on admission in south-west border of China.

T2D associated with ketosis presents most commonly in uncontrolled hyperglycemia with or without precipitating factors [5]. In rural areas, it is commonly seen due to lack of prior diagnosis or lack of proper medical treatment after diabetes diagnosis [5]. T2D patients with ketosis differ in so many respects from the typical type 1 diabetes (T1D) patients and are not completely the same with ordinary T2D patients without ketosis [6]. In this study, we examined the clinical characteristics of diabetic inpatients in order to find the same difference in clinical, biochemical, and chronic complications in T2D patients with ketosis compared to ordinary T1D and T2D patients. Furthermore, we aimed to provide health professional regional data for improvement of medical care quality in diabetic patients.

## 2. Methods

We retrospectively collected data from 3129 diabetic inpatients (1563 males and 1566 females) aged 12 years or older in the endocrinology department of Baoshan People's Hospital of Yunnan province from January 2011 to July 2015. Those with surgery, serious trauma, pregnancy, and secondary or pancreatic exocrine diseases were excluded. Patients with unconsciousness were also excluded. There were 3 groups in our study: T2D with ketosis (T2DK), T1D, and T2D without ketosis groups. Diabetes was diagnosed according to diagnostic criteria of American Diabetes Association [7]. Overweight and obesity were defined by body mass index (BMI) ≥24 (standard criteria for China set forth by the Chinese Obesity Working Group) [8, 9]. Patients were classified to T1D if they had repeated C-peptide deficiency and positive diabetes associated autoantibodies or are dependent on insulin treatment at follow-up. Patients were assigned to the T2D group if they were overweight, managed with oral hypoglycemic agents, or noncompliant with drug treatment after diabetes diagnosis. T2DK group was diagnosed according to T2D clinical and metabolic features (BMI and age at presentation, etc.) in combination with preserved *β* cell function and positive urine ketone body results. Subjects with new diagnosed diabetes or not more than 6 months after onset were recognized as new-onset. All the enrolled patients had no history of secondary diabetes. Urinary ketone body was detected using sodium nitroprusside method (Arkray Factory Inc.). Ketosis positive was diagnosed with urinary acetoacetate increased over 15 mg/dL. Plasma glucose was measured by hexokinase method. C-peptide was measured by chemiluminescent immunometric method (Roche Cobas e601). Hemoglobin A1c (HbA1c) was detected by Bio-Rad D10 automatic HbA1c analyzers. Lipids were measured by using Siemens ADVIA1800. Data were collected on clinical presentations (age, gender, family history of diabetes, height, weight, etc.) and biological parameters including plasma glucose, total cholesterol, triglycerides, HbA1c, C-peptide, and serum creatinine. All of the blood samples were performed once at the time on admission in a fasting state except for the 2 h plasma glucose and C-peptide. The chronic diabetic complications were evaluated during the hospitalization.

All data were analyzed using JMP 9.0 (SAS Institute, Cary, NC). The ANOVA test, Kruskal-Wallis test, and Mann-Whitney test were used for statistical analysis according to continuous or categorical variables. The data were expressed as means ± SD. Two-tailed *P* values <0.05 were considered significant.

## 3. Results

Among total 3129 patients, 371 (12%), 104 (3%), and 2654 (85%) patients were categorized as T2DK, T1D, or ordinary T2D (without ketosis) groups. 3084 (>98%) patients enrolled were of Han nationality and 45 patients of other origins.  Table 1 showed the demographic and laboratory data of all the enrolled subjects. The age of patients in T2DK groups (49 ± 13 yrs) was significantly older than T1D patients (26 ± 14 yrs, *P* < 0.0001), and similar to T2D patients (47 ± 14 yrs, *P* = 0.85). Male proportion was higher in T2DK (66%) group compared to T1D (44%) and T2D (48%) group. The family history was positive in about 35% T2DK patients, which was similar (*P* = 0.89) to T2D, but predominantly higher (*P* = 0.03) than T1D. BMI at admission was higher in T2DK group (25 ± 4 kg/m^2^) compared with that in T1D (19 ± 3 kg/m^2^, *P* < 0.0001), while similar to that in ordinary T2D (23 ± 3 kg/m^2^, *P* = 0.76). Significantly more patients with overweight or obese patients were in the group of T2DK (49.1%) and T2D (43.5%). There were no differences in systolic blood pressure and diastolic blood pressure among the three groups at the time of admission.

Patients of T2DK showed remarkably elevated plasma fasting glucose (16.9 ± 6 versus 10.3 ± 4 mmol/L, *P* < 0.0001 T2Dk versus T2D) and HbA1c (13.3 ± 3.1 versus 10.2 ± 2.1%, *P* = 0.01 T2Dk versus T2D) level compared to T2D upon admission. Fasting C-peptide (0.65 ± 0.2 nmol/L) in T2DK was similar to that in T1D (0.47 ± 0.1 nmol/L, *P* = 0.11), and slightly lower than that in T2D (0.98 ± 0.9 nmol/L, *P* = 0.08). However, the postprandial 2-hour C-peptide was significantly higher in T2DK (2.04 ± 0.8 nmol/L) than that in T1D (0.69 ± 0.1 nmol/L, *P* = 0.001), and similar to that in T2D (2.18 ± 1.0 nmol/L, *P* = 0.75). The patients of T2D with ketosis group had significantly higher plasma triglyceride (4.0 ± 4.0 mmol/L) than patients of ordinary T2D without ketosis group (2.8 ± 2.7 mmol/L, *P* < 0.0001), and similar to T1D group (4.3 ± 3.0 mmol/L, *P* = 0.47). Meanwhile, the total plasma cholesterol was similar in T2DK compared with T1D and T2D (5.3 ± 1.7, 5.7 ± 1.7, and 4.9 ± 1.6 mmol/L, resp., in T2DK, T1D, and T2D, *P* = 0.48 T2DK versus T1D; *P* = 0.11 T2DK versus T2D).

Diabetic patients experienced reduction of body weight on admission. For those new-onset T2DK, weight reduction was 7.3 ± 4.4 kg, significantly higher than T1D (2.1 ± 2.3 kg, *P* = 0.02) and T2D (4.5 ± 5.3 kg, *P* = 0.04) groups. For those old diagnosed diabetic subjects in T2DK, weight reduction was 9.2 ± 6.3 kg, less than T1D (15.2 ± 7.2 kg, *P* = 0.0014), and similar to T2D (10.4 ± 6.1 kg, *P* = 0.79) groups (Table 2). Moreover, among patients with existing diabetes in the T2DK group, most patients (83%) had seldom or never received regular drug treatment for diabetes, and nearly no one (<1%) had adequate glycemic control (data not shown).

**Figure 1:**
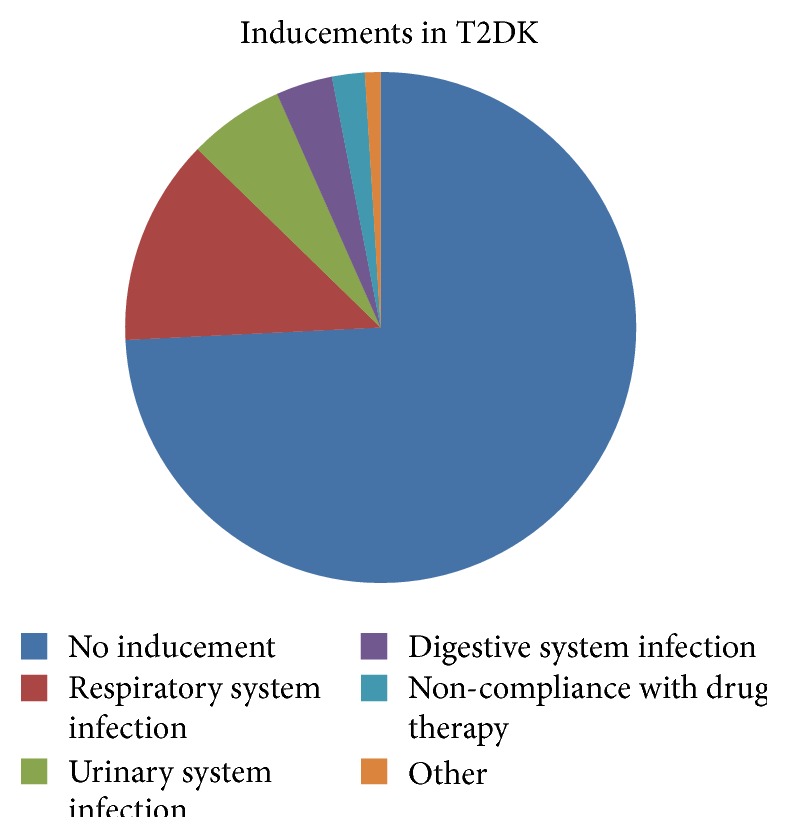
Inducements in type 2 diabetes with ketosis.

Unlike most of the T1D patients who were cases of spontaneous ketosis, in the group of T2DK, most patients were admitted with obvious inducements. The provoked ketosis was 13.8% in new-onset and 38.7% in the previously diagnosed T2D (Table 2). The predominant factors were infections. Among them, respiratory, urinary, and digestive system infections were frequent inducements in T2DK. (Figure 1).

**Figure 2:**
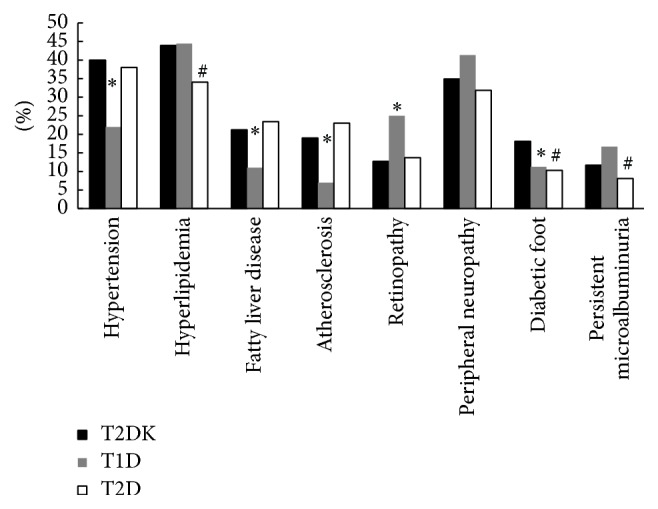
Chronic complications of diabetes. T2DK, type 2 diabetes with ketosis; T1D, type 1 diabetes; T2D, type 2 diabetes. ^*∗*^ *P* < 0.05, T2DK versus T1D; ^#^ *P* < 0.05, T2DK versus T2D.

The patients with T2DK had more atherosclerosis (19% versus 7%, resp., in T2DK and T1D, *P* < 0.0001) and fatty liver disease (21% versus 11%, resp., in T2DK and T1D, *P* < 0.0001) compared with those with T1D. Retinopathy was less in T2DK compared to T1D (12.7% versus 25%, *P* = 0.02 T2DK versus T1D). There were more cases of persistent microalbuminuria on admission in the T2DK (11.7%) compared with T2D (8.1%, *P* = 0.01). No significant differences were found in peripheral neuropathy among T2DK (34.9%), T1D (41.3%), and T2D (31.8%). The histogram of the diabetic chronic complications was demonstrated (Figure 2).

## 4. Discussion

In this study, we showed characteristics of T2D with ketosis comparing with those ordinary T2D and T1D in a tertiary hospital in Baoshan, Yunnan province of China. The T2D patients with ketosis had prominent characteristics of overweight and positive family history of diabetes. Prior to their admission, the blood glucose of most type 2 diabetes with ketosis patients in our study was poorly controlled, as reflected by elevated HbA1c levels. HbA1c could be used to determine the average blood glucose levels over 2 to 3 months. High HbA1c levels indicated the previously undiagnosed or poorly controlled diabetes.

Patients with type 2 diabetes were susceptible to ketosis or ketoacidosis under long-term uncontrolled hyperglycemia especially with inducement conditions such as infections, surgery, or trauma. Most type 2 diabetic patients irregularly or never got treatment after their first diabetic diagnosis in poor regions. Infections were common inducements in T2D with ketosis in these study subjects. In rural areas, patients with hyperglycemia were delayed for diagnosis and treatment because of the backward of economy, education, and medicine.

Type 2 diabetes with ketosis was characterized by marked hyperglycaemia, ketosis (or even ketoacidosis), and severe insulin deficiency [10, 11]. Accumulating evidence has shown that not only severe glucotoxicity but also lipotoxicity might contribute to the *β* cell dysfunction [12–16]. Our study indicated that patients in T2DK group had significantly higher plasma triglyceride than those in ordinary T2D group, which was consistent with previous study [17]. It was concluded from our study that male and overweight patients were prone to ketosis and these data confirmed the results of previous studies [14, 18]. Mechanisms underlying the reason why males were susceptible to ketosis in type 2 diabetes population were not clear up to now. A large population study [19] reported that adherence of diabetes medications was significantly associated with sex (male versus female, odds ratio 1.14, *P* < 0.0001), which might be an important reason why male predominated in T2DK group. At the same time, it has been suggested that some factors including body fat distribution, hormonal factors, and differences in the lifestyle such as alcohol abuse and smoking habits might contribute to the gender difference [14, 18, 20]. In our study, 35% male T2D ketosis patients had the habits of smoking or drinking, while the figure in female is 0.8% (data not shown).

Chronic diabetic complications were commonly seen in these study subjects, which were true with some previous studies [21, 22]. We found that T2D with ketosis patients had a similar risk of chronic diabetic complications with ordinary T2D in some aspects. However, T2D patients with ketosis were prone to suffer from diabetic foot and persistent microalbuminuria compared to ordinary T2D patients. The possible reason might be the long-term worse glucose control in the T2DK than in T2D group in our study groups. Further prolonged and large scale population studies were needed to gain definite conclusions.

There were some limitations in this report. First, the study subjects were not available for plasma ketosis test, which was more sensitive and specific than urine test. Second, this was a single-center study with limitations of study number and region; thus, the multicenter studies should be performed to further clarify the characteristics of diabetes in the south-west rural parts of China.

In summary, T2D with ketosis group occupied a large proportion on admission in total study patients. These subjects were in need of tighter glucose control due to the severe chaos of glucose and lipids metabolism and more chronic complications. The results indicated that diabetes has become a public health problem even in rural areas of China. More efforts such as intensive health education and medical resources targeted at the prevention and treatment of diabetes are urgently needed in the rural population in Yunnan province, China.

## Figures and Tables

**Table 1:** Anthropometric and biochemical characteristics of participants.

	T2DK	T1D	T2D	*P*1	*P*2
Subjects (%total)	371 (12%)	104 (3%)	2654 (85%)	—	—
Age (yr)	49 ± 13	26 ± 14	47 ± 14	<0.0001	0.85
Gender (male %)	66	44	48	<0.0001	<0.0001
Family history (%)	35	11	37	0.03	0.89
Height (cm)	162 ± 9	150 ± 11	158 ± 8	0.04	0.23
Weight (kg)	64.6 ± 14	45.3 ± 9.7	57.4 ± 11	<0.0001	0.21
Body mass index (kg/m^2^)	25 ± 4	19 ± 3	23 ± 3	<0.0001	0.76
Overweight or obese (%)	49.1	11.7	43.5	<0.0001	0.62
Systolic pressure (mmHg)	120 ± 21	123 ± 28	120 ± 27	0.41	0.87
Diastolic pressure (mmHg)	79 ± 12	77 ± 18	80 ± 14	0.83	0.92
Fasting glucose (mmol/L)	16.9 ± 6	20.1 ± 6	10.3 ± 4	0.0003	<0.0001
Postprandial 2-hour glucose (mmol/L)	24.1 ± 7	30.2 ± 7	18.4 ± 6	0.0002	0.0001
Fasting C-peptide (nmol/L)	0.65 ± 0.2	0.47 ± 0.1	0.98 ± 0.9	0.11	0.08
Postprandial 2-hour C-peptide (nmol/L)	2.04 ± 0.8	0.69 ± 0.1	2.18 ± 1.0	0.001	0.75
Hemoglobin A1c (%)	13.3 ± 3.1	14.1 ± 4.7	10.2 ± 2.1	0.43	0.01
Fructosamine (mmo/L)	3.82 ± 0.8	3.79 ± 0.9	3.14 ± 0.7	0.91	0.08
Ketoacidosis (%)	12.1	65.4	—	<0.0001	—
Total cholesterol (mmo/L)	5.3 ± 1.7	5.7 ± 1.7	4.9 ± 1.6	0.48	0.11
Triglyceride (mmo/L)	4.0 ± 4.0	4.3 ± 3.0	2.8 ± 2.7	0.47	<0.0001
Serum creatinine (*μ*mol/L)	85.6 ± 34.1	77.1 ± 38.5	80.2 ± 38	0.73	0.83

T2DK, type 2 diabetes with ketosis; T1D, type 1 diabetes; T2D, type 2 diabetes; *P*1, T2DK versus T1D; *P*2, T2DK versus T2D.

**Table 2:** Comparison of new-onset and old diagnosed diabetes.

	T2DK	T1D	T2D	*P*1	*P*2
*Duration*					
New-onset (months)	1.73 ± 2.5	0.67 ± 0.5	2.1 ± 3.2	<0.0001	0.75
Old diagnosed (years)	6.3 ± 5.3	4.4 ± 6.2	10.1 ± 9.0	0.73	0.02
*Triggers of diabetic ketosis (%)*					
New-onset	13.8	5.0	—	0.01	—
Old diagnosed	38.7	20.0	—	0.02	—
*Weight reduction (kg)*					
New-onset	7.3 ± 4.4	2.1 ± 2.3	4.5 ± 5.3	0.02	0.04
Old diagnosed	9.2 ± 6.3	15.2 ± 7.2	10.4 ± 6.1	0.0014	0.79

T2DK, type 2 diabetes with ketosis; T1D, type 1 diabetes; T2D, type 2 diabetes; *P*1, T2DK versus T1D; *P*2, T2DK versus T2D.
